# The novel mineralocorticoid receptor antagonist finerenone attenuates neointima formation after vascular injury

**DOI:** 10.1371/journal.pone.0184888

**Published:** 2017-09-19

**Authors:** Jochen Dutzmann, Robert-Jonathan Musmann, Marco Haertlé, Jan-Marcus Daniel, Kristina Sonnenschein, Andreas Schäfer, Peter Kolkhof, Johann Bauersachs, Daniel G. Sedding

**Affiliations:** 1 Dept. of Cardiology and Angiology, Hannover Medical School, Hannover, Germany; 2 Global Drug Discovery, Cardiology Research, Bayer AG, Wuppertal, Germany; Universidad de la Laguna, SPAIN

## Abstract

**Background:**

The novel nonsteroidal mineralocorticoid receptor (MR) antagonist finerenone holds promise to be safe and efficient in the treatment of patients with heart failure and/or chronic kidney disease. However, its effects on vascular function remain elusive.

**Purpose:**

The aim of this study was to determine the functional effect of selective MR antagonism by finerenone in vascular cells *in vitro* and the effect on vascular remodeling following acute vascular injury *in vivo*.

**Methods and results:**

*In vitro*, finerenone dose-dependently reduced aldosterone-induced smooth muscle cell (SMC) proliferation, as quantified by BrdU incorporation, and prevented aldosterone-induced endothelial cell (EC) apoptosis, as measured with a flow cytometry based caspase 3/7 activity assay.

*In vivo*, oral application of finerenone resulted in an accelerated re-endothelialization 3 days following electric injury of the murine carotid artery. Furthermore, finerenone treatment inhibited intimal and medial cell proliferation following wire-induced injury of the murine femoral artery 10 days following injury and attenuated neointimal lesion formation 21 days following injury.

**Conclusion:**

Finerenone significantly reduces apoptosis of ECs and simultaneously attenuates SMC proliferation, resulting in accelerated endothelial healing and reduced neointima formation of the injured vessels. Thus, finerenone appears to provide favorable vascular effects through restoring vascular integrity and preventing adverse vascular remodeling.

## Introduction

Whereas acute myocardial infarction incidence has decreased globally throughout the last two decades, the prevalence of ischemic heart failure and diabetes without or with kidney disease has steadily increased [1]. Direct deleterious effects of aldosterone and mineralocorticoid receptor (MR) activation occur in both the heart and kidneys [2]. MR blockade prevents some of these detrimental effects and markedly improves morbidity and mortality of patients with moderate to severe heart failure as evidenced by large randomized controlled clinical multi-center trials [3–6]. De Boer and colleagues showed that MRA use markedly increased over the last 20 years among patients with diabetic kidney disease, who are at high risk for vascular complications [7]. However, the available (steroidal) MR antagonists (MRAs) spironolactone, and its sole successor eplerenone, suffer from substantial drawbacks that limit their clinical use, e.g. hyperkalemia especially in patients with severe chronic kidney disease (CKD) [8]. A novel non-steroidal MRA, finerenone, has been developed in an effort to overcome these limitations by achieving high specificity for the MR as well as a balanced and equal tissue distribution between cardiac and renal tissues which is in contrast to steroidal MRAs. [9, 10]. The phase 2a MinerAlocorticoid Receptor antagonist Tolerability Study (ARTS) indeed confirmed a reduced risk for developing hyperkalemia in patients hospitalized for worsening chronic heart failure treated with finerenone compared with those treated with spironolactone despite comparable reduction of efficacy parameters like the brain natriuretic peptide (BNP), NT-proBNP, and albuminuria [11]. Moreover, in the phase 2b MinerAlocorticoid Receptor antagonist Tolerability Study-Heart Failure (ARTS-HF) the investigators found a lower incidence of the clinical composite endpoint (all-cause death, cardiovascular hospitalization or emergency presentation for worsening chronic heart failure) among patients treated with finerenone compared with eplerenone, even though the study was not powered for this observation [12].

Ischemic cardiomyopathy as a result of coronary artery disease is the leading cause for heart failure. Notably, overactivation of the MR has also been implicated in vascular remodeling processes following vascular injury in animal studies as well as in coronary artery disease and in-stent restenosis in clinical settings: Aldosterone has not only been shown to promote medial cell proliferation by direct effects on the smooth muscle cell (SMC)-MR, but to be an independent predictor for in-stent restenosis and mortality in patients with coronary artery disease [13–15]. In consequence, the effect of MRAs on vascular function and remodeling processes is of pivotal interest. Existing data on beneficial or detrimental vascular effects of spironolactone and eplerenone are inconsistent [16–18]. Based on the favorable vascular effects of MR knockout studies on the one hand [13], and the high specificity of finerenone for the MR and its unique tissue distribution profile in comparison to steroidal MRAs on the other hand, we aimed to assess the effects of finerenone on vascular remodeling processes.

## Material and methods

### Reagents

**Fig 1:**
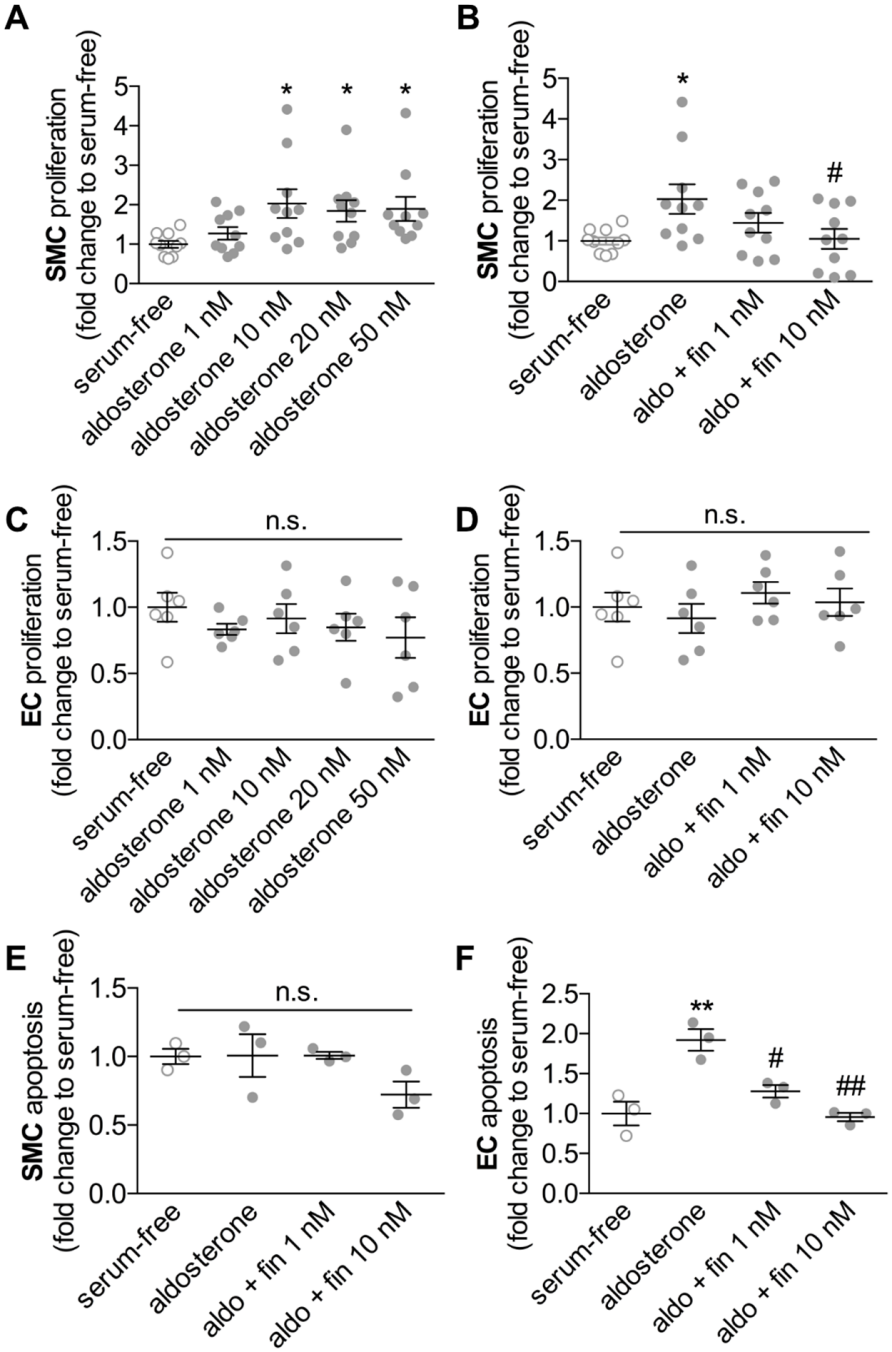
Functional effects of finerenone *in vitro*. Human smooth muscle cells (SMC) and human endothelial cells (EC) were incubated either with aldosterone alone or with aldosterone and different concentrations of finerenone, each dissolved in dimethylsulfoxide (DMSO, final concentration 0.1%) for 24 hours. A-D, Cell proliferation was determined by BrdU incorporation assays (n = 10 for SMCs/n = 6 for ECs, *P<0.05 to serum-free, #*P*<0.05 to DMSO by ordinary 1way ANOVA followed by multiple comparisons using the Tukey method). E-F, Apoptosis was determined by flow-cytometry-based caspase 3/7 activity measurement (n = 3, **P<0.01 to serum-free, #*P*<0.05 and ##*P*<0.01 to DMSO by ordinary 1way ANOVA followed by multiple comparisons using the Tukey method, aldosterone 10 nM was used for B and D-F).

Aldosterone was purchased from Sigma-Aldrich (St. Louis, MO, USA). Finerenone was provided by Bayer Pharma AG (Wuppertal, Germany). For *in vitro*-studies, aldosterone or finerenone were dissolved in dimethylsulfoxide (DMSO, Cat. W387520, Sigma-Aldrich). For oral application in *in vivo*-studies, finerenone was dissolved in 40% macrogol (15)-hydroxystearate (Solutol^®^, Cat. 42966, Sigma-Aldrich) and 10% ethanol. *In vitro*, aldosterone was used at concentrations of 10 nM except for Fig 1A and 1C, where aldosterone was used at indicated concentrations.

### Cell culture

Human coronary artery smooth muscle cells (SMC) and human umbilical vein endothelial cells (EC) were purchased from Lonza (Cologne, Germany). Cells between passages 2 and 4 were used for all experiments and cultured in optimized growth media according to the supplier’s protocols.

Cells were incubated with aldosterone with or without finerenone for 24 hours after 24 hours of serum-starvation for the assessment of cell proliferation and apoptosis. Immediately prior to the addition of aldosterone, cells were preincubated with finerenone or vehicle for 30 minutes.

### Functional in vitro assays

Quantification of cell proliferation was assessed by using a BrdU-based Cell Proliferation ELISA according to the manufacturer’s protocol (Cat. 11 647 229 001, Roche Applied Science, Mannheim, Germany). Cell apoptosis was quantified by using a FLICA^®^ 660 caspase 3/7 assay kit according to the manufacturer’s protocol (Cat. 9152, ImmunoChemistry Technologies, Bloomington, MN, USA).

### Vascular injury models

All procedures concerning animal experiments complied with the Directive 2010/63/EU of the European Parliament as well as with local ethical guidelines and had been approved by the Lower Saxony’s institutional committee for animal research (LAVES). Adult male C57BL/6 mice were purchased from Charles River (Sulzfeld, Germany).

#### 1.1.1. Mouse carotid artery model of reendothelialization

The electric deendothelialization of the carotid artery was performed as previously described [19]. Briefly, mice were anesthetized by a singular intraperitoneal injection of 100 mg/kg body weight ketamine hydrochloride (Anesketin, Albrecht, Aulendorf, Germany) and 16 mg/kg body weight xylazine (Rompun^®^ 2%, Bayer Health Care AG, Leverkusen, Germany) diluted in 0.9% sodium chloride. Adequate anesthesia was confirmed by the lack of tail-pinch-induced pain reflex. The left common carotid artery was exposed through ventral middle line neck incision and injured with a bipolar microregulator (ICC50, ERBE-Elektromedizin GmbH, Tuebingen, Germany) below the carotid bifurcation. An electric current of 2 W was applied for the duration of 2 seconds to each millimeter of the carotid artery over a total length of 4 mm with the use of a size marker parallel to the artery. Immediately before surgery and then once daily, finerenone or vehicle was delivered as oral gavage. Three days after carotid injury, reendothelialization was evaluated by staining of the denuded area after injection of 50 μL of a 5% Evan’s blue solution. Pictures of *en face* prepared injured arteries were taken and reendothelialization was assessed. The reendothelialized area was calculated as difference between the blue-stained area and the initially injured area by computer-assisted morphometric analysis (ImageJ 1.48 software, National Institutes of Health, Bethesda, MD, USA) and presented as percentage of reendothelialization.

#### 1.1.2. Mouse femoral artery injury model of neointimal hyperplasia

The dilation of the femoral artery was performed as previously described [20, 21]. In brief, mice were anesthetized as described above. For the wire-induced injury model of the femoral artery, a straight spring wire (0.38 mm in diameter, Cook Medical Inc., Bloomington, IN, USA) was advanced through the profunda femoris artery for 1 cm into the femoral artery and left in place for 1 minute. After withdrawal, the profunda femoris artery was ligated and reperfusion of the dilated femoral artery was confirmed. Immediately before surgery and then once daily, finerenone or vehicle was delivered as oral gavage. At 21 days after dilation, mice were sacrificed, blood was drawn from the right ventricle, and perfusion with PBS or 4% para-formaldehyde (PFA, Carl Roth, Karlsruhe, Germany) in PBS was performed via the left ventricle. The femoral artery was carefully excised and postfixed in 4% PFA and embedded in Tissue-Tek OCT embedding medium (Sakura Finetek Europe B.V., Zoeterwoude, The Netherlands). Afterwards, the arteries were snap-frozen and stored at -80°C until sectioning.

### Morphometry

The whole femoral artery was cut in 6 μm serial sections and 6 cross-sections from regular intervals throughout the artery were stained with van Gieson staining (n = 6 mice per condition). For morphometric analyses, ImageJ 1.48 software was used to measure external elastic lamina, internal elastic lamina, and lumen circumference, as well as medial and neointimal area.

### Immunofluorescence

Femoral artery cross sections or cell samples were incubated with antibodies recognizing α-SMA (C6198, Sigma-Aldrich) or Ki-67 (ab15580, Abcam plc). Ensuing incubations were carried out with Alexa 488-coupled secondary antibodies (LifeTechnologies) and counterstained with nuclear 4.6-diamidino-2-phenylindole (Immunoselect Antifading Mounting Medium DAPI, Dianova GmbH, Hamburg, Germany). Monoclonal antibodies to α-SMA were labelled directly with Cy3. Negative controls were conducted by substituting the primary antibody through an appropriate species- and isotype-matched control antibody (Santa Cruz Biotechnology).

### Microscopy

Tissue samples were analyzed using bright-field and immunofluorescence microscopy (Eclipse TE2000-S, Nikon Instruments Europe B.V., Amstelveen, The Netherlands) equipped with appropriate filter blocks and image processing software (NIS Elements AR 4.20.01, Nikon Instruments Europe B.V.,).

### Statistical analysis

Data were stored and analyzed on personal computers using Microsoft Excel 2010 (Microsoft Corporation) and GraphPad Prism 6.01 (GraphPad Software Inc., La Jolla, CA, USA). Data among study groups were analyzed by ordinary one-way ANOVA or 2way ANOVA followed by pair wise multi comparisons using the Tukey method depending on the number of groups and affecting factors. All data are represented as mean ± standard error of the mean (SEM). A probability value <0.05 was considered statistically significant for all comparisons.

## Results

### Finerenone prevents aldosterone-induced EC apoptosis and SMC proliferation in vitro

To investigate vascular cell function in response to aldosterone with or without finerenone *in vitro*, EC and SMC were incubated with different concentrations of aldosterone and finerenone. At 24 hours after stimulation, we detected significantly increased SMC proliferation rates following stimulation with 10 nM, 20 nM or 50 nM aldosterone as assessed by BrdU-incorporation assays. Whereas finerenone treatment at concentrations of 1 nM showed a clear trend towards reduced SMC proliferation rates, 10 nM finerenone sufficiently and significantly prevented aldosterone-induced SMC proliferation (*P<0.05 to serum-free, ^#^*P*<0.05 to DMSO, n = 6, Fig 1A and 1B). However, aldosterone did not affect EC proliferation *in vitro*, and there was also no effect of finerenone (Fig 1C and 1D).

In contrast, flow cytometry-based detection of FLICA^®^-labeled SMC revealed no aldosterone-dependent induction of SMC apoptosis (Fig 1E). In contrast, EC apoptosis was increased after stimulation with aldosterone *in vitro* but this effect could be prevented by the treatment with finerenone even at low concentrations of 1 nM (**P<0.01 to serum-free, ^#^*P*<0.05 and ^##^*P*<0.01 to DMSO, n = 6, Fig 1F).

### Finerenone accelerates the re-endothelialization process following vascular injury

**Fig 2:**
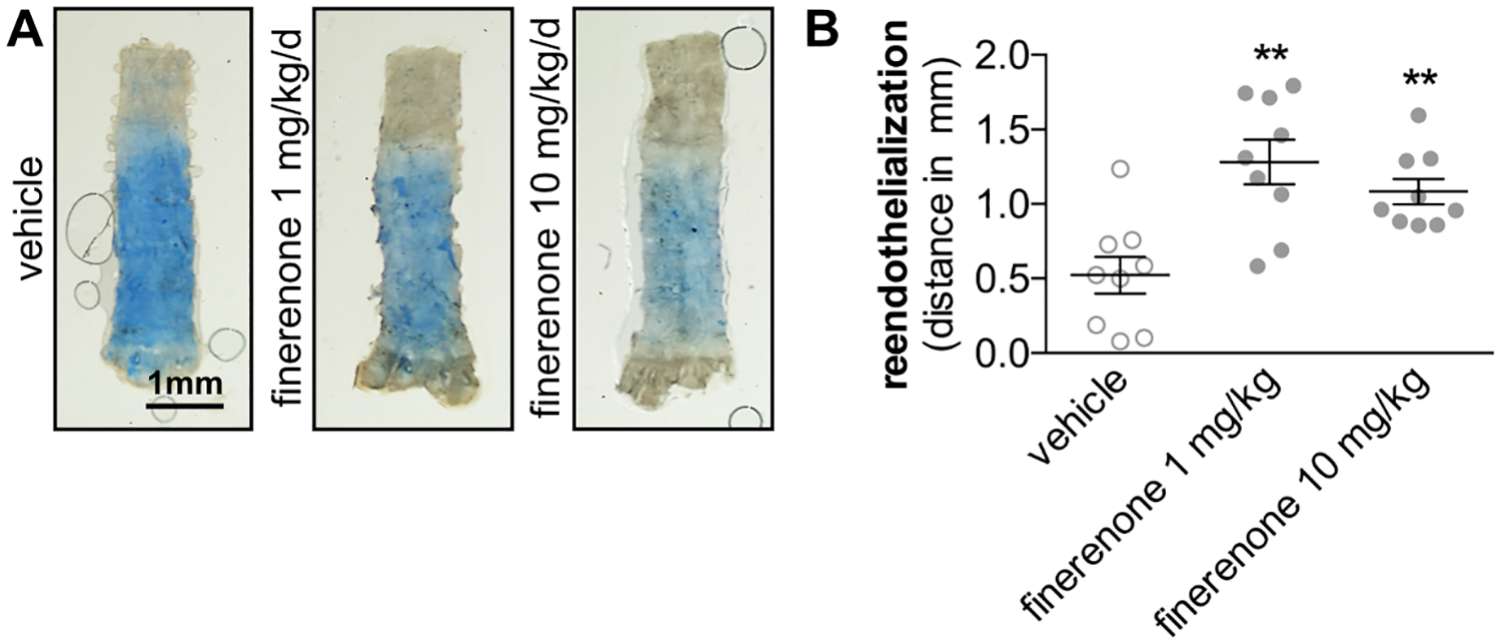
Finerenone promotes early endothelial recovery. Electrical denudation of the carotid artery was performed in 10 weeks old C57BL/6J mice. Finerenone or vehicle was daily delivered as oral gavage. A, Three days following injury, endothelial regeneration was evaluated by injection of a 5% Evan’s blue solution and en face microscopy. B, The re-endothelialized distance was calculated by substraction of the deendothelialized distance from 4 mm (standardized denudated area, n = 9, **P<0.01 by ordinary 1way ANOVA followed by multiple comparisons using the Tukey method).

Early endothelial recovery was assessed by Evan’s blue injection and en face microscopy 3 days after electric injury of the carotid artery in C57BL/6 mice. Daily oral application of finerenone (1 mg/kg/d or 10 mg/kg/d) markedly accelerated the re-endothelialization process at that time point compared with daily vehicle application (0.52 ± 0.12 mm re-endothelialization in vehicle-treated mice vs. 1.13 ± 0.16 mm in 1mg/kg/d finerenone-treated mice vs. 1.083 ± 0.086 mm in 10 mg/kg/d finerenone-treated mice, **P<0.01 to vehicle, n = 8, Fig 2).

### Finerenone reduces the recruitment of leukocytes and the inflammatory response following vascular injury

**Fig 3:**
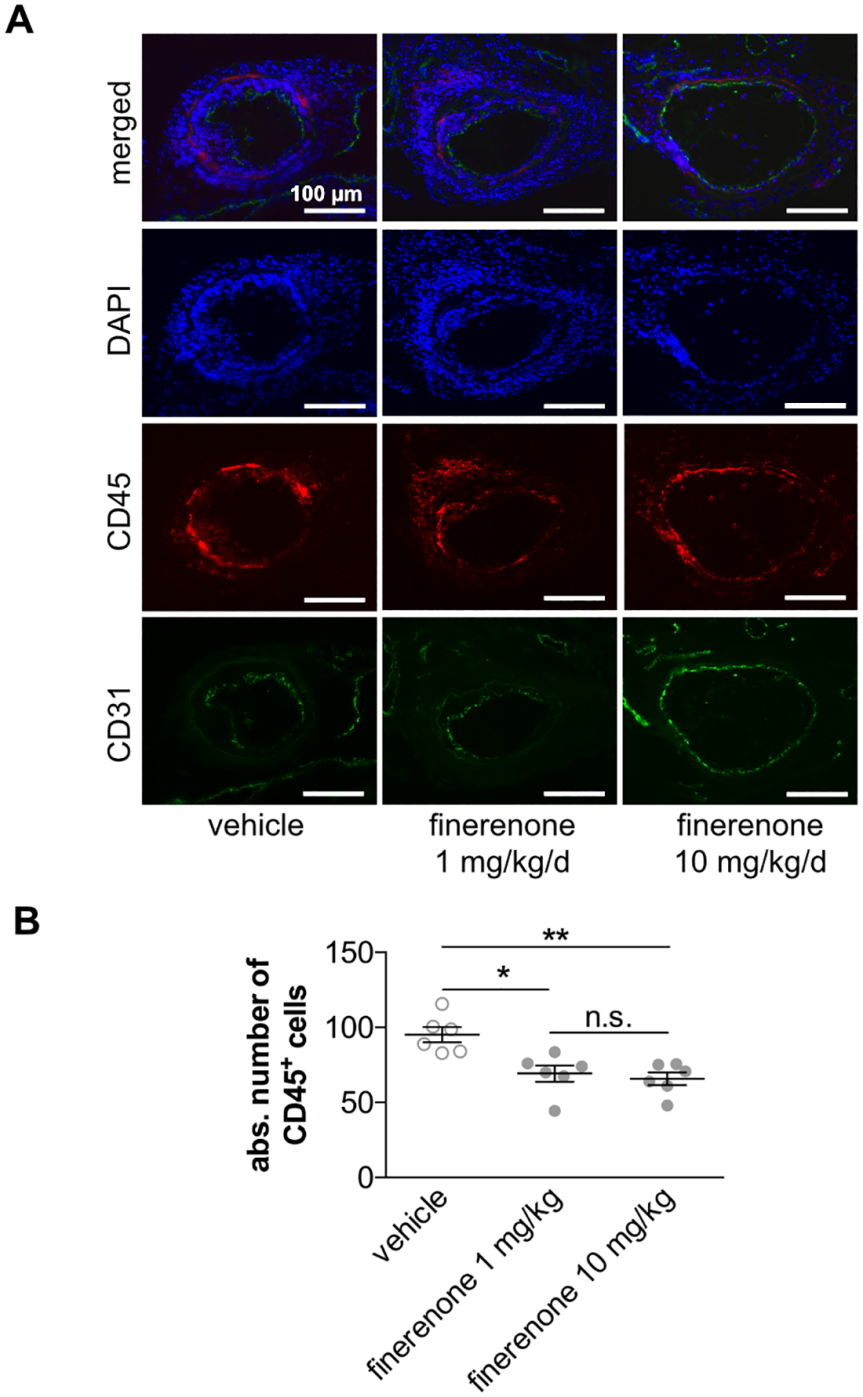
Finerenone reduces the intimal and medial leukocyte content. Wire-induced femoral artery dilation was performed in 10-week-old C57BL/6 mice. Finerenone or vehicle was daily delivered as oral gavage. A, Ten days after injury, leukocyte content was assessed by immunfluorescence staining for the pan-leukocyte marker CD45 (red). Co-immunostaining for CD31 (green) and staining of nuclei with DAPI (blue) was performed to assess the endothelial lining and the overall cell number for better morphological orientation and to allow quantification. B, The amount of leukocytes was determined as the total number of CD45^+^ cells (n = 6, *P<0.05, **P<0.01 by ordinary 1way ANOVA followed by multiple comparisons using the Tukey method).

The number of accumulating leukocytes in vascular lesions was determined by immunohistochemical detection of the pan-leukocyte marker CD45 at 10 days following wire-induced injury of the murine femoral artery. Oral application of finerenone dose-dependently and significantly reduced the amount of leukocytes within both the intimal and the medial vascular layer (95.14 ± 5.07 in vehicle-treated mice vs. 66.33 ± 8.13 in 1 mg/kg/d finerenone-treated mice vs. 65.69 ± 4.26 in 10 mg/kg/d finerenone-treated mice, *P<0.05, **P<0.01, n = 6, Fig 3.

### Finerenone attenuates smooth muscle cell proliferation and neointimal lesion formation following vascular injury

**Fig 4:**
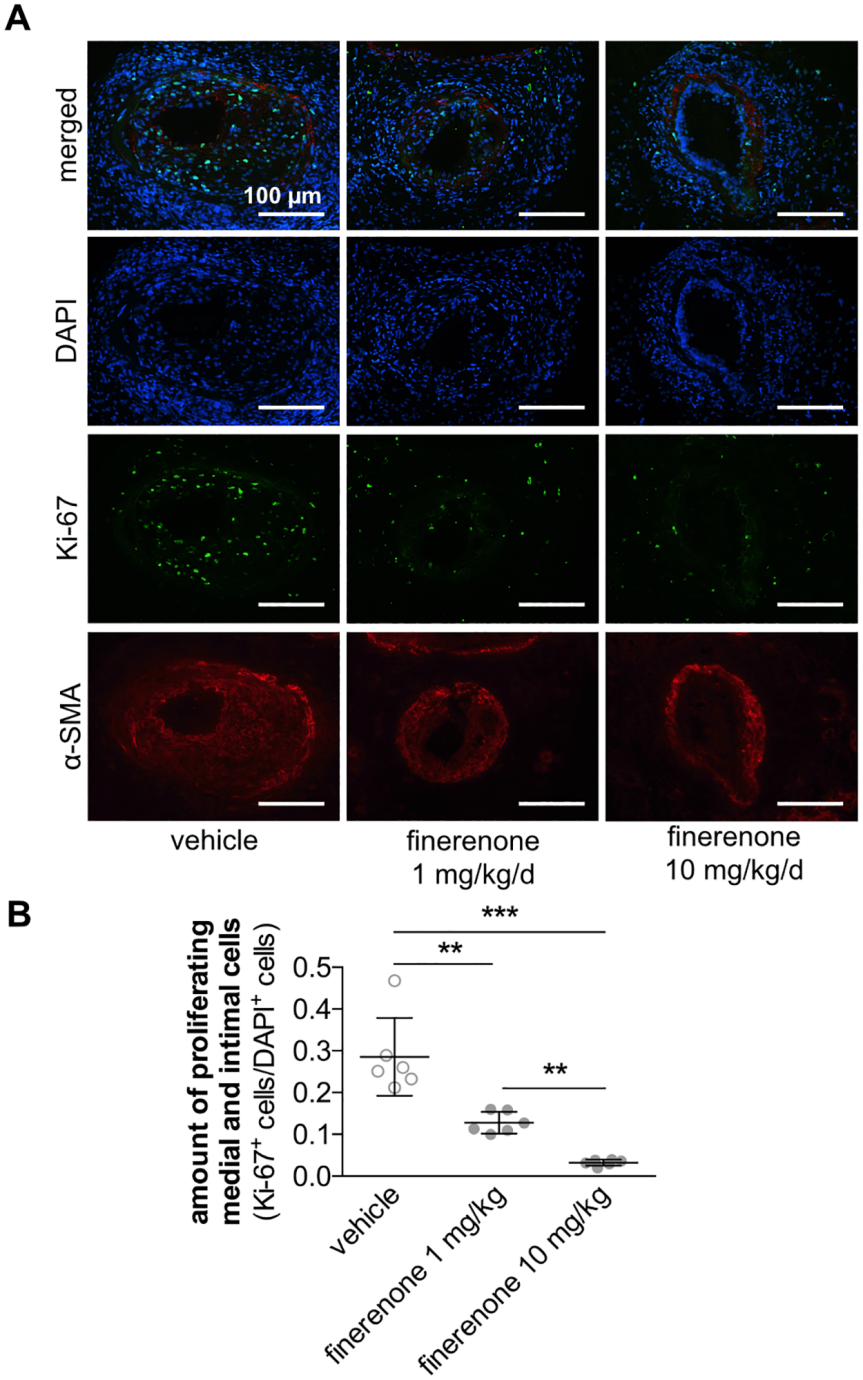
Finerenone prevents medial and intimal cell proliferation. Wire-induced femoral artery dilation was performed in 10-week-old C57BL/6 mice. Finerenone or vehicle was daily delivered as oral gavage. A, Ten days after injury, cell proliferation was assessed by immunfluorescence staining for DAPI (blue), α-smooth muscle actin (α-SMA, red), and Ki-67 (green). B, The amount of proliferating cells was determined as Ki-67^+^ cells/DAPI^+^ cells (n = 6, **P<0.01, ***P<0.001 by ordinary 1way ANOVA followed by multiple comparisons using the Tukey method).

**Fig 5:**
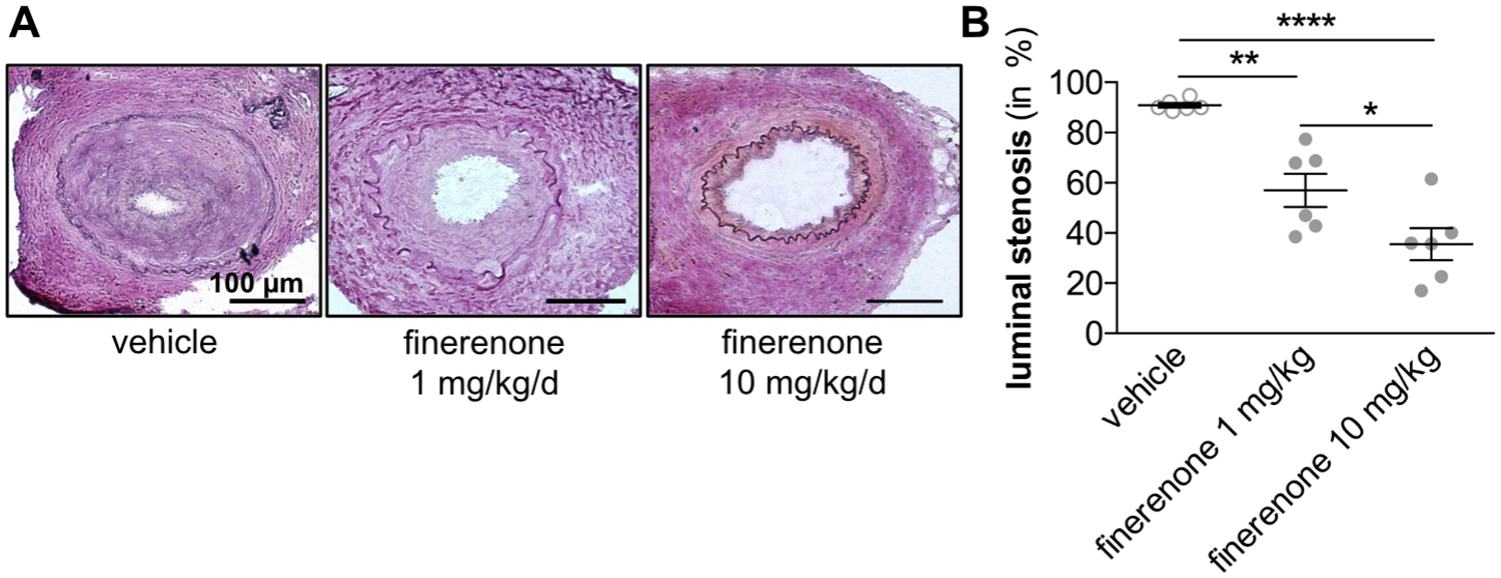
Finerenone attenuates neointima lesion formation. Wire-induced femoral artery dilation was performed in 10-week-old C57BL/6 mice. Finerenone or vehicle was daily delivered as oral gavage. A, 21 days after injury, neointimal lesion formation was assessed by van Gieson staining. B, Luminal stenosis was calculated as percent stenosis = [1 − (A_L_/A_N_)] × 100, A_L_ = luminal area, and A_N_ = area of the normal artery defined as the area surrounded by internal elastic lamina (n = 6, *P<0.05, **P<0.01, ****P<0.0001 by ordinary 1way ANOVA followed by multiple comparisons using the Tukey method).

Intimal and medial cell proliferation was determined by immunohistochemical staining for the proliferation marker Ki-67 10 days following wire-induced injury of the murine femoral artery. Oral application of finerenone dose-dependently and significantly reduced the amount of proliferating Ki-67^+^ cells within both the intimal and the medial vascular layer (ratio of Ki-67^+^/DAPI^+^ cells 0.281 ± 0.032 in vehicle-treated mice vs. 0.127 ± 0.011 in 1 mg/kg/d finerenone-treated mice vs. 0.032 ± 0.002 in 10 mg/kg/d finerenone-treated mice, **P<0.01, ***P<0.001, n = 6, Fig 4). Conclusively, formation of a neointimal lesion was significantly impaired in mice treated with 1 mg/kg/d finerenone 21 days after injury. This effect could be further augmented by application of 10 mg/kg/d finerenone (luminal stenosis 90.84 ± 0.922% in vehicle-treated mice vs. 57.02 ± 6.630% in 1 mg/kg/d finerenone-treated mice vs. 35.50 ± 6.340% in 10 mg/kg/d finerenone-treated mice, *P<0.05, **P<0.01, ****P<0.0001, n = 6, Fig 5).

Peripheral blood samples 10 and 21 days after injury did not indicate any significant difference between the vehicle-treated group and the finerenone-treated group in regard to electrolyte metabolism, liver- or kidney function (Table 1). Most importantly, there was no increase in plasma potassium with the use of finerenone, in fact, there was rather a trend for a decrease in plasma potassium especially with the lower finerenone dose.

**Table 1:** Blood values in mice treated with vehicle or finerenone.

	ref. values [22]	vehicle	finerenone 1 mg/kg/d	*P* value	finerenone 10 mg/kg/d	*P* value
Potassium [mmol/l]	3.1–6.1	5.1±0	4.33±0.16	n.s.	4.6±0.2	n.s.
Sodium [mmol/l]	149–165	163±2	163.00±2.00	n.s.	164±0.00	n.s.
Chlorid [mmol/l]	n/a	104±2	107.33±1.11	n.s.	102±3	n.s.
creatinine [μmol/l]	28–11	8.5±2.5	6.67±0.44	n.s.	12±0.00	n.s.
Urea [mmol/l]	3.2–13.2	11.4±0.7	11.871.42	n.s.	10.65±0.65	n.s.

## Discussion

Atherosclerotic vascular disease is the leading cause for heart failure. Thus, the impact of (novel) therapeutics for the treatment of heart failure on vascular remodeling processes is of fundamental interest. Inhibitors of the renin-angiotensin-aldosterone system (RAAS) have been shown to be not only cardio protective but in addition exhibit particular nephro protective effects in patients with diabetic kidney disease [5, 7]. Whereas certain evidence exists on favorable vascular effects of inhibitors of the angiotensin-converting enzyme (ACE) [23], findings on the influence of steroidal MRAs on vascular remodeling processes are inconsistent.

Here, we provide evidence that the highly specific novel non-steroidal MRA finerenone prevents aldosterone-induced SMC proliferation and EC apoptosis *in vitro*. *In vivo*, oral application of finerenone significantly accelerates the re-endothelialization process and thus limits leukocyte recruitment at the site of injury, and reduces the proliferation of SMC and neointimal lesion formation in mice.

Very recently, results from the ARTS-HF study verified beneficial effects of finerenone in the treatment of patients with chronic heart failure who also have diabetes mellitus and/or chronic kidney disease. In this high-risk population, finerenone exerted a good safety profile comparable with that of eplerenone but, in contrast, significantly reduced the composite end point of death from any cause, cardiovascular hospitalizations, or emergency presentations for worsening heart failure [12]. Moreover, the MR has been shown to be crucially involved in early myocardial healing processes after coronary artery ligation in mice [24], and treatment with finerenone resulted in improved left ventricular compliance as well as reduced interstitial fibrosis compared with control mice following myocardial infarction [25]. Our study now shows for the first time that finerenone may not only be beneficial in sufficiently treating heart failure or improving myocardial healing, but also in preventing vascular remodeling processes.

The underlying molecular signaling mechanisms responsible for the distinct effects of finerenone in vascular cells remain not well defined. However, the relative instability of the MR *in vitro*—as soon as vascular cells are removed from their native surrounding—has challenged previous attempts to further elucidate the underlying MR-dependent mechanisms [26]. Moreover, recent evidence for profound paracrine effects, which are dependent on intact MR-signaling, underlines the importance to study the impact of MRAs in intact organisms and tissues *in vivo* [27].

Mechanistically, well-conducted *in vivo* studies in animals with tissue-specific MR knockout indicated several possible underlying molecular processes: Vascular SMC-specific MR knockout decreased SMC proliferation and prevented pathological vascular remodeling in a wire-induced carotid injury model through a placental growth factor/type 1 vascular endothelial growth factor receptor pathway [13]. Notably, this conditional knockout also reduced oxidative stress in EC in a paracrine manner [25]. EC-specific MR knockout improved endothelial cell function in a mouse-model of western diet-induced endothelial dysfunction due to reduced oxidative stress and an increased anti-inflammatory polarization of macrophages [28]. Finally, selective deletion of the MR in myeloid cells has very recently been shown to limit macrophage accumulation and vascular inflammation following vascular injury through impaired nuclear factor-κB (NF-κB) signaling, thus preventing neointimal hyperplasia [29]. Given the distribution to the vascular space as well as well perfused organs and considering the MR selectivity of finerenone, finerenone-mediated vascular effects may predominantly involve these signaling pathways validated in genetically modified mouse models [25].

The high MR potency and selectivity combined with its physicochemical properties which lead to its unique tissue distribution profile may also be the reason for the clear and robust positive effect of finerenone on EC- and SMC function and neointima formation *in vivo* observed in this study [30]. In contrast, only inconsistent effects of spironolactone or eplerenone on vascular function were reported. Further studies will have to clarify the possibly distinct effects of the different classes of clinically available MRAs on vascular cell functions. Moreover, large animal studies or further clinical observations will be needed to confirm the positive effects of finerenone on vascular remodeling processes that were observed in this study.

## Conclusions

The novel selective non-steroidal MRA finerenone promotes endothelial healing and inhibits neointimal lesion formation following vascular injury. Thus, in addition to its beneficial effects in heart failure therapy, finerenone might provide favorable vascular effects through restoring vascular integrity and preventing adverse vascular remodeling following percutaneous coronary interventions. This might be particularly important for the treatment of patients with ischemic cardiomyopathy due to coronary artery disease.

## Supporting information

S1 File ARRIVE guidelines checklist. A completed copy of the ARRIVE guidelines checklist, a document that aims to improve experimental reporting and reproducibility of animal studies for purposes of post-publication data analysis and reproducibility, is provided as supporting information.
(DOCX)

S2 File Minimal data set. Raw data of all figures are provided as supporting information (Excel file).
(XLSX)

## Abbreviations

ACE: angiotensin-converting enzyme
ARTS: MinerAlocorticoid Receptor antagonist Tolerability Study
ARTS-HF: MinerAlocorticoid Receptor antagonist Tolerability Study-Heart Failure
BNP: brain natriuretic peptide
CKD: chronic kidney disease
EC: endothelial cell
MR: mineralocorticoid receptor
MRA: mineralocorticoid receptor antagonist
RAAS: renin-angiotensin-aldosterone system
SMC: smooth muscle cell
