# Supplementary material for: The novel mineralocorticoid receptor antagonist finerenone attenuates neointima formation after vascular injury
Source: PLoS One. 2017 Sep 19;12(9):e0184888. doi: 10.1371/journal.pone.0184888 (PMC5605005; doi:10.1371/journal.pone.0184888)

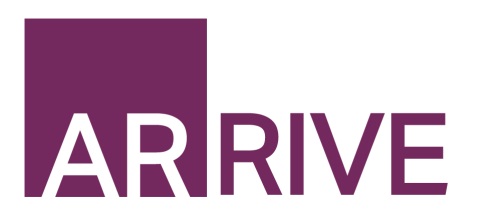


The ARRIVE Guidelines Checklist

Animal Research: Reporting In Vivo Experiments

Carol Kilkenny^1^, William J Browne^2^, Innes C Cuthill^3^, Michael Emerson^4^ and Douglas G Altman^5^

*^1^The National Centre for the Replacement, Refinement and Reduction of Animals in Research, London, UK, ^2^School of Veterinary Science, University of Bristol, Bristol, UK, ^3^School of Biological Sciences, University of Bristol, Bristol, UK, ^4^National Heart and Lung Institute, Imperial College London, UK, ^5^Centre for Statistics in Medicine, University of Oxford, Oxford, UK.*

|  | | ITEM | RECOMMENDATION | Section/ Paragraph |
| --- | --- | --- | --- | --- |
| 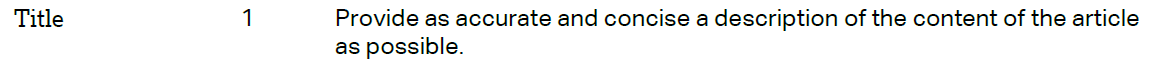 | | | Title |  |
| 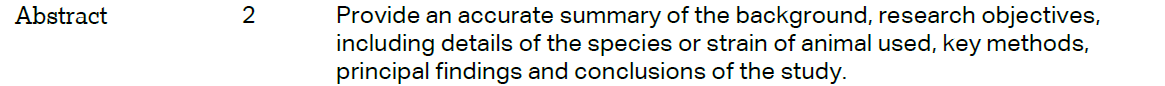 | | | Abstract |  |
| INTRODUCTION | | |  |  |
| 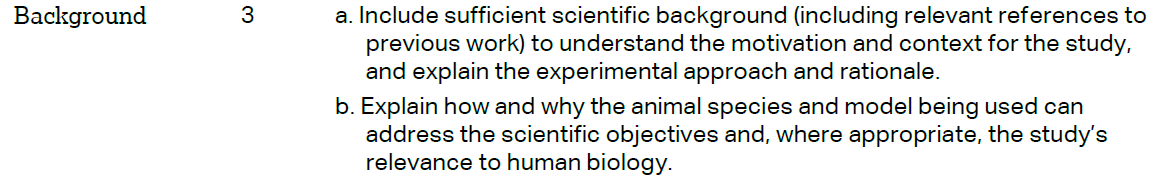 | | | Section 1  Section 1 and sections 2.4.1-2.4.2 |  |
| 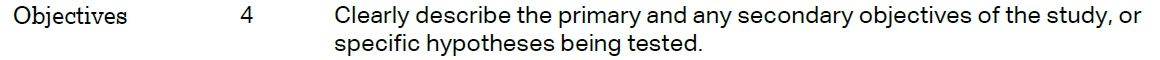 | | | Section 1 |  |
| METHODS | | |  |  |
| 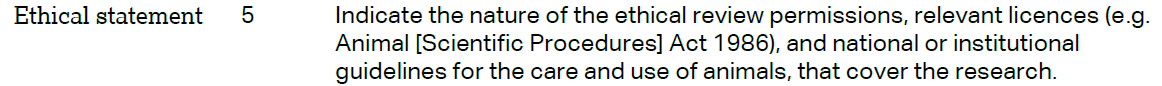 | | | Section 2.4 |  |
| 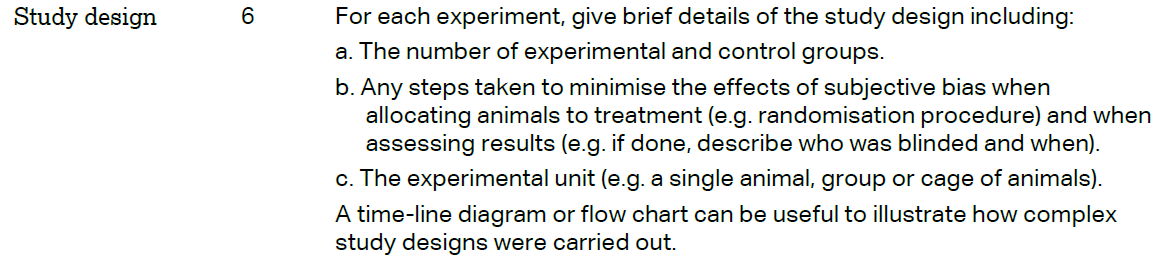 | | | Figures 2-5  Section 2.4  Sections 2.4.1-2.4.2 |  |
| 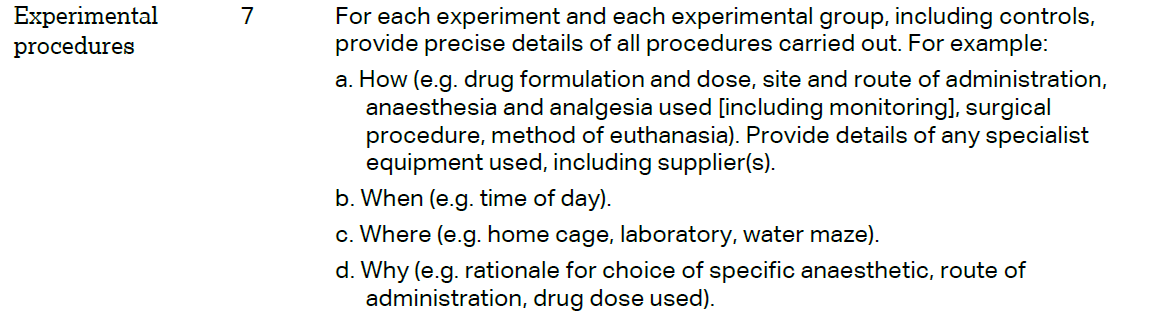 | | | Sections 2.4.1-2.4.2 |  |
| 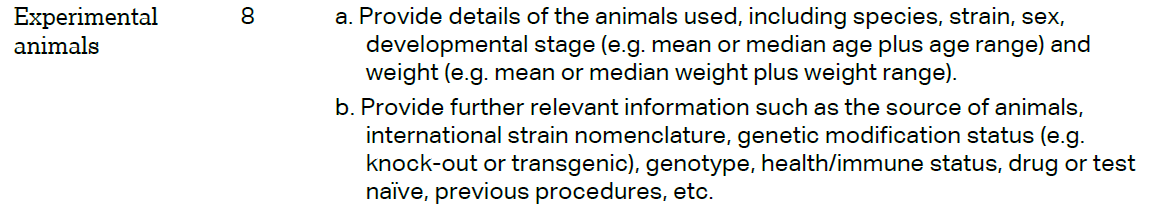 | | | Section 2.4 |  |

The ARRIVE guidelines. Originally published in *PLoS Biology*, June 2010^1^

| 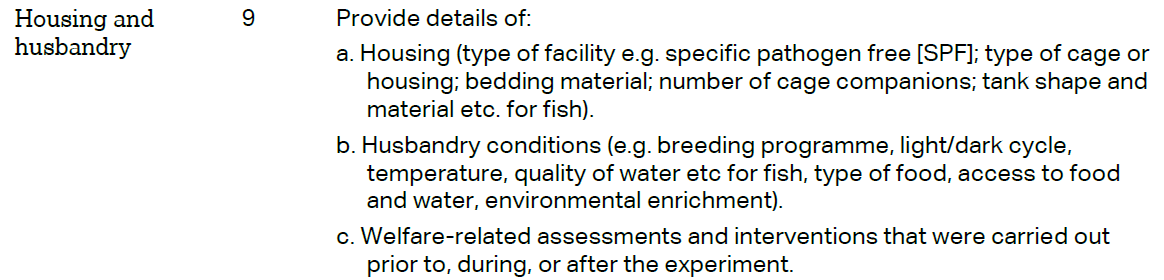 | Sections 2.4.1-2.4.2 | |
| --- | --- | --- |
| 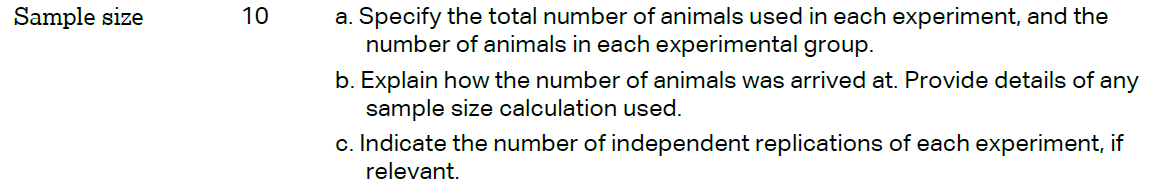 | Figures 2-5  Section 2.4 | |
| 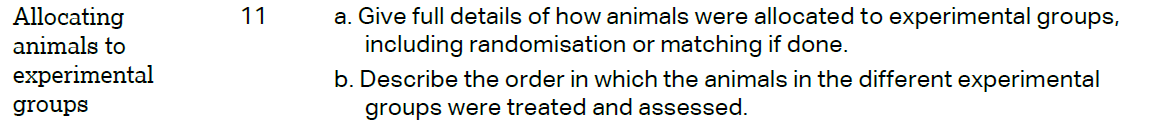 | Sections 2.4.1-2.4.2 | |
| 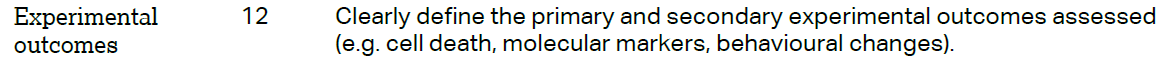 | Section 3  Figures 2-5 | |
| 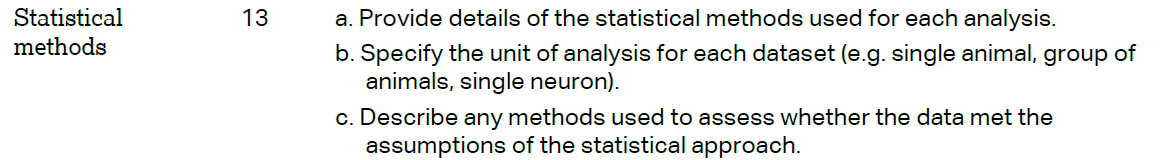 | Section 2.8  Figures 2-5 | |
| RESULTS |  | |
| 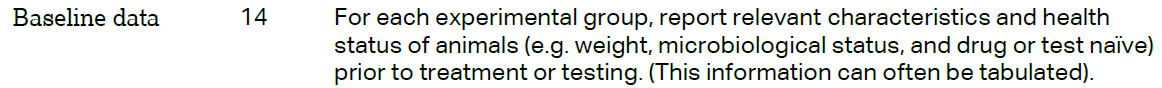 | Section 2.4 | |
| 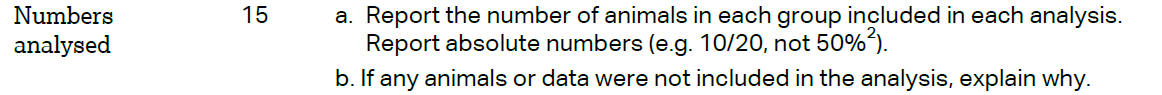 | Figures 2-5 | |
| 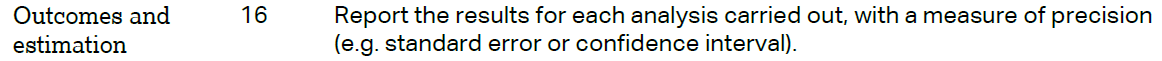 | Figures 2-5 | |
| 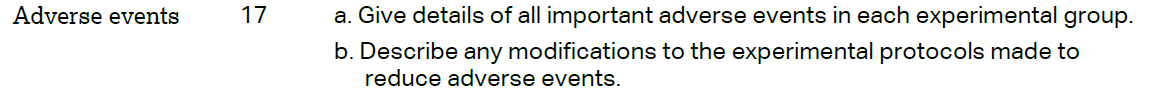 | Section 2.4 | |
| DISCUSSION |  | |
| 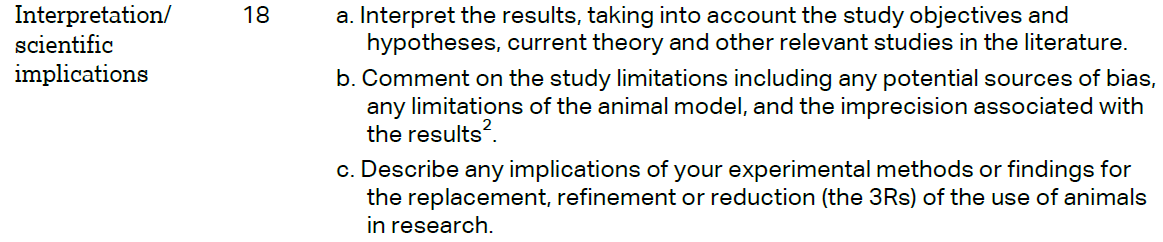 | Sections 3 and 4  Section 4  Section 2.4 | |
| 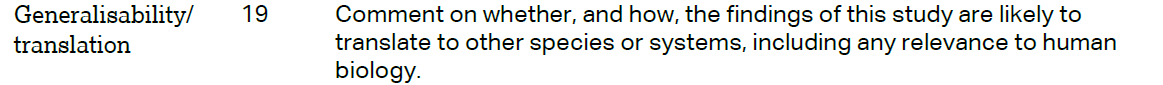 | Section 4 | |
| 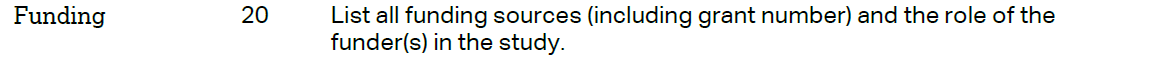 | | Section “Funding “ |


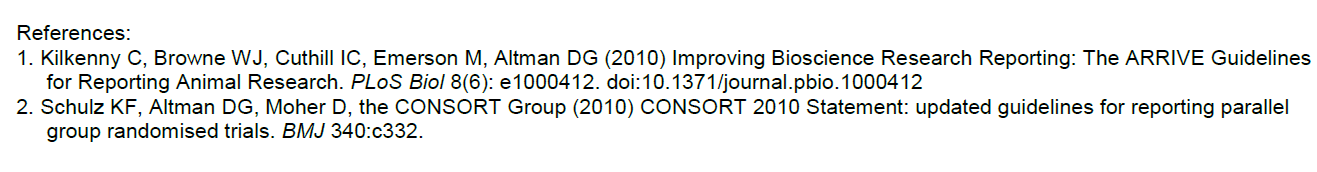

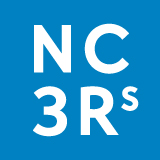

Supplement: S1 File — A completed copy of the ARRIVE guidelines checklist, a document that aims to improve experimental reporting and reproducibility of animal studies for purposes of post-publication data analysis and reproducibility, is provided as supporting information. (DOCX) [file pone.0184888.s001.docx]
